# Anesthetics

**Published:** 1852-06

**Authors:** 


Art. VII.—*Anesthetics.* Medical Examiner for May 1852.

British and Foreign Medico-Chirurgical Review, for January, 1852.

At a meeting of the Philadelphia County Medical Society, held on the 13th of April, an interesting discussion took place on the merits of anesthetics, and as the subject is one upon which the minds of professional men are still far from being accordant, we propose transferring to our pages some of the testimony for and against these remarkable agents brought out on that occasion; and if our limits permit, we shall take a more general survey of the state of public opinion in reference to anesthesia. If not, we may return to the subject at an early day. Dr. Patterson, one of the members, who had lately returned from Europe, was requested to give the society the result of his observation on the use of ether and chloroform; upon which he replied that:

He had until recently felt, as the great body of the profession in Philadelphia feel, in regard to the anesthetics. His position had been that of doubt, of distrust, and avoidance of them as a dangerous innovation. These agents came to us in a very questionable shape originally. When the first report of anesthesia in surgery reached us from Boston, it came, not only startling us by its novelty and the magnitude of the change in practice it contemplated, but also shocking us by its violation of our ethical notions and the savor of empiricism that hung about it. The new agent had a new, high-sounding and unscientific name, and there were rumors of a patent-right to be secured to its discoverers. But when it was shown to be plain sulphuric ether, which any chemist could manufacture and any practitioner administer—when the name of Letheon disappeared, and the letters-patent were no longer heard of—especially when indisputable evidence of its power and utility reached us, we should have given the subject a more favorable reception and examination than we have done. But the mass of resistance to the use of anesthetics has been more obstinate and impregnable here than any where else in Christendom; and our hospital—alone among great hospitals—has never permitted their employment even in the most painful operations. Participating in these feelings, and never having administered either chloroform or ether in his practice, Dr. P. visited Europe during the past summer, and one of the objects he proposed to himself was to obtain, if possible, more light upon this much mooted question.

In Edinburgh he had the opportunity of seeing and hearing a great deal of the use of chloroform, and it was in regard to the experience of that city that he desired mainly to speak. The undeniable and most important fact is, that in Edinburgh, where chloroform has been used with a degree of freedom, and with a frequency and extent elsewhere unknown, not a single unpleasant circumstance has attended its administration. Every obstetrician, surgeon and dentist in that capital uses it, without exception. Every teacher of obstetrics advises its employment, and gives it to his patients in labor, some in all cases and others only in cases of unusual duration and severity. No surgeon thinks of performing a serious or painful operation, one likely to be attended with great suffering or severe shock, without bringing his patient under its influence. Thousands of patients have inhaled it to full anesthesia ; the chemists manufacture and sell it daily in large quantities ; and no one has been injured or even made ill by it. There does not remain a practitioner in Edinburgh opposed to its use. An infinite amount of pain has been relieved, great suffering has been prevented, the dread of the operation has been in a great measure removed, and no harm has been done to a single individual. There is no denying these facts. There can be no supposition of concealment or unfair dealing. The character of the gentlemen in whose hands its employment is, of itself precludes the idea as one not to be entertained for a moment. In a large proportion of the cases, if not in all, concealment would be impossible were it attempted. Dr.Sim pson introduced the chloroform to our notice as a therapeutic agent, he bears the unqualified testimony that he has never seen it do any mischief whatever to his patients. Dr. S. is a man whose word, in a matter of medical experience, must be taken without qualification. He has not only the candor of an honest man, but also the discriminating judgment which is not readily deceived, and the calm deliberateness which is not easily led away by the enthusiasm of a hobby. By his kindness Dr. P. was enabled to witness the administration of chloroform to several patients, and to converse with others who had used it in child-birth, some for a considerable length of time. In some cases Dr. P. has maintained its effects for several hours. This powerful agent was always administered with due caution, but with a fearless hand. The patient was thrown into a condition of perfect insensibility, and underwent operations otherwise painful, without any apparent sensation, and certainly with no subsequent recollection of suffering. The manner of Dr. S. indicates the most entire confidence in both the safety and utility of his practice. The lying-in women whom Dr. S. saw were all in excellent health and spirits, and Dr. P. thought that their average " getting up" was shorter and better after chloroform than without it. All asserted that they awoke from the slumber without the slightest after-sensation or recollection of pain, without headache or vertigo or nausea, and precisely as after a refreshing sleep. One spirited and intelligent lady, with whom Dr. P. had the honor to converse, roundly asserted that the ladies of Edinburgh *would* have chloroform whether their doctors gave it to them or not; that she would not approach her confinement without it, and that the knowledge that this invaluable boon was within her reach, had taken away all the trembling apprehension which had previously made her pregnancy a period of dread and suffering.

In other parts of Great Britain and on the continent, the anesthetics are not used so freely as in Edinburgh, but still they are resorted to in all severe surgical operations, and very frequently in obstetric practice. There are very few who use them in every case of midwifery to which they are called, but there are still fewer who do not use them in some cases. No surgeon of any note refuses their employment altogether. They are used in every hospital of London and Paris, more or less. Dr. P. was amused by the ardor of a gentleman connected with St. Bartholomew's, who, in a conversation on the subject, enthusiastically declared that all the other benefits conferred on mankind by the discovery of America, (bark and our democratic liberty included,) were eclipsed by the inestimable addition of anesthetics to the treasury of the healing art.

The substance used exclusively in Edinburgh, and most generally elsewhere, is chloroform. The accounts we have of unpleasant or fatal effects from it, do not deter them from administeriug it. Dr. P. saw no sulphuric ether given, and met with no physician who employed it. The objections to its use are its highly stimulating qualities, (increasing the risk of fever and hemorrhage,) its unpleasant and exceedingly persistent odor, and its liability to leave a disturbed condition of the stomach with gastric uneasiness and acid eructations. The question of danger from chloroform he would not discuss at present, except to express his conviction that it is ascribable, in all cases, to some error in the method of administration or in the management of the patient or of the inhaling apparatus. Dr. Simpson gives it on a napkin or handkerchief, lightly folded in a funnel shape and held near the mouth. In obstetrical cases he gives it on the approach of each pain, removing the napkin the moment the relaxation of the patient's muscles shows that the pain is passing off, and not reapplying it until her uneasy motions indicate the approach of another pain and that it is beginning to be felt.

In view of all these circumstances, Dr. P. inquired whether there is not reason to believe that we in Philadelphia have been a little unreasonable in our opposition to these agents. Certain it is, that they alleviate much suffering for which we have hitherto had no remedy, and that they have in a measure taken away the curse of child-birth, and deprived surgical operations of much of their horror.

Dr. Condie, in the following extract, embodies the whole force of the argument against anesthetics.

Dr. Condie was inclined to believe, that a very considerable change, in the opinion of European practitioners as to the employment of anesthetics, had recently taken place. That a much greater degree of caution in their administration was now inculcated, and that the circumstances under which a resort to them solely to prevent pain, is proper, are admitted to be much more restricted than was the case upon their first introduction. This he inferred, from the very different tone assumed, of late, by the medical journals of Europe when treating of anesthetics and of the cases in which their administration is justifiable. Much of the wild enthusiasm by which the propriety of a resort to them, in almost every case attended with pain or suffering, was insisted upon by their early advocates, have disappeared. It is now admitted, on all hands, that the leading anesthetic (chloroform) is an active poison, and that to prevent disastrous consequences following its administration, the utmost caution must be observed ; that we are not always able to determine the effects that may be produced, in different individuals, by the inhalation of the same quantity of the article , that the induction of anesthesia is not justifiable in trifling operations, or in ordinary cases of perfectly natural labor ; that, even in those cases in which anesthesia is considered proper during parturition, it is unnecessary to push it to the extent of inducing an entire abolition of consciousness; and finally, that there are a number of circumstances the presence of which forbids absolutely, in all cases, a resort to it.

Anesthetics have been in use for too short a period to enable us to arrive at any very certain conclusions in regard to their real value as therapeutic agents—to determine accurately all the cases and circumstances to which they are adapted, or to lay down the proper rules for their safe administration. It unfortunately happens that, whenever any important remedy is announced, instead of a dispassionate examination of its properties, and a cautious testing of its curative powers, it is at once seized upon by a set of enthusiasts, who laud it far beyond its deserts, and ascribe to it remedial virtues under circumstances where it is afterwards ascertained to be powerless for good, if its effects be not even positively injurious; and if the agent be one of great activity, it is only after the infliction of much injury from its injudicious employment, that it attains its proper place upon the lists of the materia medica. When iodine was introduced as a remedy, to believe those who first described its properties and therapeutic powers, it was to remove, at once, consumption from the list of incurable diseases, and to arrest all scrofulous affections, with as much certainty as the bark or quinine is known to arrest the paroxysms of intermittent fever.

But, in reference to no remedial agent, has this wild enthusiasm been displayed to as great an extent perhaps, as it has in regard to anesthetics. By their agency pain was to be expunged from the catalogue of human evils—child-bearing was to be divested of its agony, and the knife of the surgeon was to remove the diseased member from the body without the patient suffering a single pang, and this without the slightest danger or inconvenience either immediate or remote.

No wonder need be experienced that anesthesia should have secured from the very first so many ardent advocates, that it should have been recklessly resorted to in almost every operation, in every case of labor, and in almost every disease attended with pain or acute suffering of any kind. Nor has, as yet, the occurrence of death after death, directly attributable to the influence of the agents employed in in its production, been a sufficient warning to deter all from a resort to these, merely for the prevention or relief of pain.

By the physician, pain is to be studied with very different feeling from those prompted by a morbid sensibility. Its removal may not, under all circumstances, be advisable. Though invariably an evil, still, cases may certainly occur, in which it would be better to allow the patient to endure, for a short period, even severe suffering, than to induce in him a state of complete insensibility. It was the opinion of Dr. Physick, that, generally speaking, important operations are more successful in those patients who give full vent to their feelings, than in those, who, by a powerful effort of the will, suppress all indications of suffering. And there is some foundation for the inquiry whether, even admitting that, by the employment of anesthetics, surgical operations may be divested of pain without any immediate danger to the life of the patient, the ultimate result of such operations is as favorable, upon the whole, as of those in which anesthesia has not been induced.

A comparison that has been made by an acute and accurate observer, in our midst—based on the statistics of several large hospitals—of the results of operations in the same institution, before and after the introduction of anesthetics, as well as the results of operations performed in those hospitals where anesthetics are now invariably employed, with that of the same class of operations performed in the Pennsylvania Hospital, without the induction of anesthesia, would lead us to suspect that the abolition of pain by anesthetic agents does actually exert an unfavorable influence upon the result of operations; an opinion at which Dr. Porter of the U. S. army had arrived from his experience of the use of these agents, during the war with Mexico.

In obstetrics, Dr. Ramsbotham, in the last edition of his *Obstetric Medicine and Surgery*, has denounced the employment of anesthetics, excepting in some few exceptional cases; while Dr. Chowne, in a late discussion before the London Medical Society, remarked, that he had seen most disastrous consequences follow the administration of chloroform—not only simple after-consequences, but serious mental disorders. The fact of the occurrence of insanity from the use of chloroform is insisted upon by Dr. Webster, who has adduced several cases in which such a result occurred; his statements are of a character that forbid our treating them with entire indifference.

But even if we could, with positive and invariable safety, prevent the pains of parturition by the employment of anesthetics—would it always be proper to do so? There may be some practitioners of such consummate skill in the application of obstetrical instruments, who, directed solely by their knowledge of the anatomy of the pelvis, and the position of the head, can apply them, without any risk of injury to the organs of the female, as well while she is in a state of entire unconsciousness as when she is in a condition to advise him, promptly, of any pain he may inflict upon her. Dr. Condie, however, very freely confessed that he preferred always, in the application of instruments during labor, that the susceptibility of the female to painful impressions should be unimpaired, as an additional safeguard to prevent her suffering injury from maladroitness on his part.

Much surprise has been expressed by Dr. Parrish, that the comparatively few cases in which fatal effects have resulted from the employment of anesthetics should be used as an argument against a resort to them in surgical operations and during labor, when the same objection will equally lie against the administration of any of the more potent and useful articles of the materia medica. But is it not far more surprising that the gentleman should not see the very material difference between the employment of a dangerous remedy to *save life*, and a resort to a similar remedy *merely to destroy pain*, in a case that we have every reason to believe, so far as the safety of the patient is concerned, will do very well without it? It will not do to point to the 9000 operations safely performed at St. Bartholomew's Hospital under the influence of anesthetics, in evidence of the propriety of resorting to it, as a mere preventive of pain—for the one single case of death acknowledged to have occurred in that institution from the use of anesthesia, independently of others of which we have been informed, is sufficient to counterbalance all the good supposed to have resulted from it, by the mere abolition of pain, in the 9000 cases. The life of one patient in 10,000 ought not to be sacrificed to obtain for the remaining 9999, relief from what is, in fact, in most cases, a very short period of suffering.

Dr. C. remarked, that he did not wish to be considered as altogether opposed to the employment of anesthetics as therapeutic agents. He was satisfied that their introduction had armed us with a most potent and valuable means of relief in a numerous class of cases. From their internal use, by inhalation, as well as from their application externally, he has derived the most beneficial results in many instances: he has seen them arrest, immediately, the most violent paroxysms of puerperal convulsions, allay promptly the intense suffering induced by cramp of the stomach and intestines, and that the attendant upon neuralgic affections of the internal organs, and of various portions of the surface. He could even conceive of operations in which the risk of danger from their employment should not deter us from availing ourselves of their pain-abolishing effects. An operation may be necessary to save life, but one, at the same time, attended with so much and so long continued suffering, as to cause the patient to hesitate between its endurance and the *certainty of death* should it be omitted or delayed. In such a case, Dr. C. would not hesitate to recommend the induction of anesthesia—believing that the risk the patient will incur from it will be fully compensated by its enabling him to undergo an operation, the pain of which it would, otherwise, be scarcely worth enduring to purchase the few additional years of existence it may possibly bestow. Cases too occur, not necessarily fatal, of violent paroxysms of pain, occurring periodically, at shorter or longer intervals, and during a very long period of time, where relief from suffering, by which life is rendered a burden, is cheaply purchased by any remedy, even one, the exhibition of which may be attended with considerable danger. Such cases, are, unquestionably, proper ones for the employment of anesthetics; under many other circumstances also, they will no doubt be found useful and judicious remedies—even although no other good effect should attend their exhibition than the relief of pain.

In reply to Dr. Condie, Dr. Parrish brought forward other statistics. In a word, we think he completely answers Dr. Condie's objections:

Dr. Parrish remarked that he was surprised to find the statistics of Hospitals introduced here as opposed to anesthesia in large surgical operations. The basis of the calculation, as I understand it, is a comparison between the results of amputations in the hospitals of New York and Boston, since the introduction of anesthetic agents into these institutions, and those of the Pennsylvania Hospital, where they are not used. Dr. Condie, who generally speaks with such accuracy, on questions when facts are concerned, has not brought forward the figures by which he claims to sustain his position, but has made a general statement that a comparison between these institutions is altogether unfavorable to the use of anesthetics.

Now, will any one undertake to say that a slight difference in the percentage of mortality, after operations of any given class, running through a period of only a few years, and without taking into account all the attending circumstances, the comparison being made between three institutions, and those not of the largest class, is to be taken as evidence against the use of an article, which has been employed thousands of times in the largest hospital establishments in the world?

Why, it is well known that the statistics of a hospital bearing upon any point, may vary very much from year to year, and that a series of unfortunate results in one or two years will influence very materially the general results for a series of years. Thus an epidemic erysipelas may prevail, and carry off a large number of patients who have been subjected to capital operations; and if the institution should be visited with such an epidemic for several seasons in succession, the mortality may become so heavy, as to keep it in the rear of the more favored institutions for a long series of years. There are, too, various other circumstances which may influence the mortality, entirely independent of the use of anesthetics, and which in my judgment would render it very unsafe to draw conclusions, based upon the experience of two or three institutions during a period of a few years. I apprehend, also, that a difference will be found in the percentage of mortality after any given capital operation, during a series of years, amongst the best hospitals of this and other countries, both before and since the introduction of anesthetic agents, showing that other causes are constantly varying the results.

But if a difference in the mortality of hospitals prior to, and since the introduction of anesthetic agents, is to be taken as an argument for or against them, we have a much more extended field of comparison than that offered by the three chief hospitals of this country.

The large hospitals of Europe furnish much more reliable data; and it so happens that the advocates of anesthesia point triumphantly to the statistics of these institutions as affording ample evidence of a diminished mortality after the great capital operations, since the introduction of these pain-destroying agents.

In the elaborate work of Professor Simpson, on anesthesia in Surgery and Midwifery, the reader will find two chapters devoted to the question, whether anesthesia increases or decreases the mortality attendant on surgical operations. The author has here collected the statistics of mortality after the large amputations, in different countries, as derived from official sources, including Phillips' table, which comprise not only the results in hospital practice, but in the private practice of physicians in Great Britain and in other countries, so far as recorded in the periodical literature of these countries. He has also availed himself of Malgaigne's tables of results from the Parisian hospitals, forming altogether one of the most complete and extensive collections of cases yet published. The percentage of mortality after large amputations, calculated from these data, which were formed prior to the introduction of anesthetic agents, are then compared with the results after the same operations with anesthetics, as derived from the British and Parisian hospitals, forming a series of upwards of 300 cases, up to the time when Professor Simpson wrote.

The conclusion arrived at is, that the mortality since the introduction of anesthesia is sensibly diminished, and the argument in favor of anesthetics thus derived is considered by Dr. Simpson as most cogent and convincing. I can only refer the members to this array of figures on the ether side of the question, and ask them to compare them with those now brought forward on the other side, and see which is the stronger.

I believe myself that there is some degree of uncertainty in this method of calculation, but if it is relied on, etherization has evidently the advantage.

In regard to the recent unfortunate death from chloroform at St. Bartholomew's hospital—it is certainly against it—though it is only one death in over nine thousand cases, in which the article has been administered there; still, this with the others which have been reported, shows that chloroform is a most potent agent, and that it should not be given on trivial occasions, and without great caution, while at the same time one death in ten thousand should not cause us to abandon it. I myself have always preferred the ether, believing it to be safe and reliable, and should not use the more powerful agent, where this answered the purpose.

Dr. Hays referred to the cases of death from anesthetics, which he said amount now to more than thirty, and remarked that he deemed the possibility of such an accident a sufficient reason why professional men should still exercise caution in the use of these agents. A fatal case has lately happened in St. Bartholomew's Hospital, London, where, up to that time chloroform had been used in nine or ten thousand instances without "a stain on its character as an innocuous agent of good." The history of this case is related in the *Examiner*, to which we are indebted for a report of this discussion, and as in some particulars it is one of much interest, we append it for the benefit of readers who may be forming their opinions on this subject. Dr. Hays thus gives it:

The subject of this case, was a young man only 23 years of age, affected with aneurism by anastomosis implicating the ear and a portion of the scalp behind it, and which had commenced when the patient was four years of age. He was admitted into St. Bartholomew's Hospital, and Mr. Lloyd determined to attempt its cure by ligating the vessels supplying it. Chloroform was given to the patient, and he was kept under its influence for half an hour, the operation having proved a tedious one. He recovered from its influence without any ill effects. The tumor was not, however, entirely obliterated. A little over a month subsequent to this operation, Mr. Lloyd found on examination an artery between the angle of the jaw and mastoid process which pulsated strongly, and on compressing it so as to arrest the circulation in it, the pulsation in the tumor entirely ceased. Mr. Lloyd determined to apply a ligature to it. Chloroform was again administered by the careful and experienced assistants of the hospital, and the article used was from the same bottle which had been administered in the previous operation. In from five to ten minutes he came under the influence of the article, and Mr. Lloyd commenced the operation. Scarcely had he incised the skin when his assistant informed him, that the patient's pulse was sinking, and before any restoratives could be applied, he ceased to breathe. Every means that could be devised were resorted to in order to re-animate him, but without success. On post-mortem examination, no morbid organic change in any organ was found to explain the fatal occurrence; but the blood appeared to have been poisoned. "It was fluid, and it remained without coagulation after its escape from the heart and vessels. It had also a brownish purple hue, much like that which is commonly observed in the spleen; none of it, when thinly spread out, presented the ordinary dark, black or crimson hue of venous blood." But for this untoward administration of chloroform, the patient might have lived to the usual term of human existence. Here, then, is a case in which no human foresight could have provided against the fatal result. The chloroform used was pure, and had been employed before without any ill effects; there was no idiosyncrasy in the patient forbiding its employment, for he had previously been under its influence with apparent impunity; that there was no want of caution and skill in its administration, we have the assurance in the fact that it was given by the experienced assistants of the Hospital; and that there was no organic disease forbiding its employment, was demonstrated by the autopsy. If this case is not calculated to prove that danger attended the administration of chloroform, Dr. Hays said he would be at a loss to know what kind of evidence gentlemen required for that purpose.

By far the most important event of the 19th century connected with medicine, unquestionably, is the practical use of anesthetics. Whatever may be the estimate of their value, no one can doubt that they have wrought great changes in the practice of the healing art. That they are a boon of inestimable value we have long been thoroughly convinced; but at the same time it would be folly to attempt to disguise the fact, that it has come to us not unmixed with evil. Anesthesia is not unattended with danger. The victims to it might possibly, now, be counted by scores. Certainly more than thirty have perished by etherization. But amid these disasters public confidence in the safety of the practice has been steadily increasing. The wonder experienced is, that so few have been killed by agents capable of exerting so powerful an influence upon the human system. No doubt they are used more cautiously for these accidents, but it can not be doubted by any one that their use is rapidly extending.

But we have no room for remarks of our own, and will only add that we regard the reputation of anesthesia as fixed and secure. If professional men were now disposed to give the practice up, they would not be permitted to do so by their patients. Anesthetics, we may be sure of that, "will never down."

